# Dosimetric Evaluation of Commercially Available Flat vs. Self-Produced 3D-Conformal Silicone Boluses for the Head and Neck Region

**DOI:** 10.3389/fonc.2022.881439

**Published:** 2022-08-10

**Authors:** Stephan Pollmann, André Toussaint, Michael Flentje, Sonja Wegener, Victor Lewitzki

**Affiliations:** University Hospital Würzburg, Würzburg, Germany

**Keywords:** flat silicone bolus, individual silicone bolus, 3D conformal silicone bolus, 3D printer, head and neck cancer, fused deposition modeling (FDM), surface dose measurement, volumetric modulated arc therapy (VMAT)

## Abstract

**Background:**

Boluses are routinely used in radiotherapy to modify surface doses. Nevertheless, considerable dose discrepancies may occur in some cases due to fit inaccuracy of commercially available standard flat boluses. Moreover, due to the simple geometric design of conventional boluses, also surrounding healthy skin areas may be unintentionally covered, resulting in the unwanted dose buildup. With the fused deposition modeling (FDM) technique, there is a simple and possibly cost-effective way to solve these problems in routine clinical practice. This paper presents a procedure of self-manufacturing bespoke patient-specific silicone boluses and the evaluation of buildup and fit accuracy in comparison to standard rectangular commercially available silicone boluses.

**Methods:**

3D-conformal silicone boluses were custom-built to cover the surgical scar region of 25 patients who received adjuvant radiotherapy of head and neck cancer at the University Hospital Würzburg. During a standard CT-based planning procedure, a 5-mm-thick 3D bolus contour was generated to cover the radiopaque marked surgical scar with an additional safety margin. From these digital contours, molds were 3D printed and poured with silicone. Dose measurements for both types of boluses were performed with radiochromic films (EBT3) at three points per patient—at least one aimed to be in the high-dose area (scar) and one in the lower-dose area (spared healthy skin). Surface–bolus distance, which ideally should not be present, was determined from cone-beam CT performed for positioning control. The dosimetric influence of surface–bolus distance was also determined on slab phantom for different field sizes. The trial was performed with hardware that may be routinely available in every radiotherapy department, with the exception of the 3D printer. The required number of patients was determined based on the results of preparatory measurements with the help of the statistical consultancy of the University of Würzburg. The number of measuring points represents the total number of patients.

**Results:**

In the high-dose area of the scar, there was a significantly better intended dose buildup of 2.45% (95%CI 0.0014–0.0477, p = 0.038, N = 30) in favor of a 3D-conformal bolus. Median distances between the body surface and bolus differed significantly between 3D-conformal and commercially available boluses (3.5 vs. 7.9 mm, p = 0.001). The surface dose at the slab phantom did not differ between commercially available and 3D-conformal boluses. Increasing the surface–bolus distance from 5 to 10 mm decreased the surface dose by approximately 2% and 11% in the 6 × 6- and 3 × 3-cm^2^ fields, respectively. In comparison to the commercially available bolus, an unintended dose buildup in the healthy skin areas was reduced by 25.9% (95%CI 19.5–32.3, p < 0.01, N = 37) using the 3D-conformal bolus limited to the region surrounding the surgical scar.

**Conclusions:**

Using 3D-conformal boluses allows a comparison to the commercially available boluses’ dose buildup in the covered areas. Smaller field size is prone to a larger surface–bolus distance effect. Higher conformity of 3D-conformal boluses reduces this effect. This may be especially relevant for volumetric modulated arc therapy (VMAT) and intensity-modulated radiotherapy (IMRT) techniques with a huge number of smaller fields. High conformity of 3D-conformal boluses reduces an unintended dose buildup in healthy skin. The limiting factor in the conformity of 3D-conformal boluses in our setting was the immobilization mask, which was produced primarily for the 3D boluses. The mask itself limited tight contact of subsequently produced 3D-conformal boluses to the mask-covered body areas. In this respect, bolus adjustment before mask fabrication will be done in the future setting.

## Introduction

In radiotherapy, the 3D printing fused deposition modeling (FDM) technique introduced by Crump (1) has been used in a variety of ways including the creation of individualized phantoms, brachytherapy applicators, or intraoral stents (2–4). The fabrication of individualized boluses *via* 3D printing represents another application of this technique.

Bolus material can effectively modify the radiation dose to the skin and mucosal surfaces (5–7). Liquid-impregnated gauzes, wax, gel, and silicone overlays are conventionally used for this purpose. In this context, a transition between bolus and skin that is as seamless as possible is crucial for the predictability of dose distribution, as even small gaps can lead to significant superficial dose reductions and dose inhomogeneity (8–12).

A literature review by Pugh et al. showed that the improved surface conformity of 3D-printed boluses could prove beneficial for volumetric modulated arc therapy (VMAT), as the presence of air gaps can result in a 10% reduction in surface dose for small field sizes and oblique incident beams (13).

Gaps under commercially available boluses are commonly observed in the head and neck region, as irregularities on the surface can lead to wrinkling or poor bolus formability. Furthermore, since VMAT with numerous small fields prone to the dose effects of increased surface–bolus distance plays a prominent role in the treatment of head and neck tumors, bolus conformity may be of importance. A simultaneous reduction of the bolus cover on healthy skin enabled by the bolus individualization may reduce side effects.

Individual boluses produced *via* 3D printing are typically made of solid plastics and have been tested in numerous dosimetric analyses (13–18). A major drawback of this design is the low flexibility and thus patient comfort. The use of new 3D printing techniques such as FDM may offer the possibility to produce cost-effective, individual, and flexible boluses for routine clinical use (19–24). Therefore, in this study, we combined FDM with a silicone casting process so that we were able to get flexible 3D-compliant boluses and implement the attachment into routine clinical workflow.

Previous studies of 3D-conformal flexible boluses did not pay attention to the dose effects outside the covered area. The merits of 3D-conformal boluses in terms of accuracy of superficial dose application have so far been demonstrated in the context of simulations and phantom measurements (8–12). This adds to the clinical advantage of adjusting the bolus configuration to the individual patient situation. A detailed investigation of economic and clinical aspects however was not planned yet.

While a comparable production process of a flexible bolus was already described (18), existing studies are limited to the evaluation by means of a static anthropomorphic phantom and recalculation using the therapy planning system (TPS) (16, 25). Further, to our knowledge, there are no reports of an evaluation of the fit accuracy for patients and the dosimetric characteristics using *in vivo* dosimetry.

The aim of this study was to investigate dose coverage of superficial target volumes (surgical scars) with the simultaneous investigation of dose reduction in surrounding healthy skin by *in vivo* dosimetry with 3D-conformal and commercially available silicone boluses. A standardized workflow for bolus production was also introduced. Radiochromic films were used to evaluate dose buildup and to quantify the influence of gaps between bolus and skin. The conformity of the 3D-printed boluses was investigated by measuring the distance between bolus and skin in the TPS.

## Materials and methods

### Equivalence of Materials

To test the equivalence of materials from conventional and individual boluses with respect to dose buildup, six measurements each were performed using MOSFET-live dosimetry on the slab phantom. For this purpose, a commercially available bolus and a flat-cast self-produced bolus were used, each with a layer thickness of 0.5 cm and an area of 30 cm × 30 cm. Irradiation was performed with the fixed number of 100 monitor units (MUs) at a focus-slab phantom distance of 100 cm in standing field technique with a nominal field size of 10 × 10 cm^2^ on a Siemens linear accelerator (model Siemens Primus Mevatron M, 6 MV).

### Influence of Bolus–Surface Distance and Field Size on Dose Reduction

**Figure 1:**
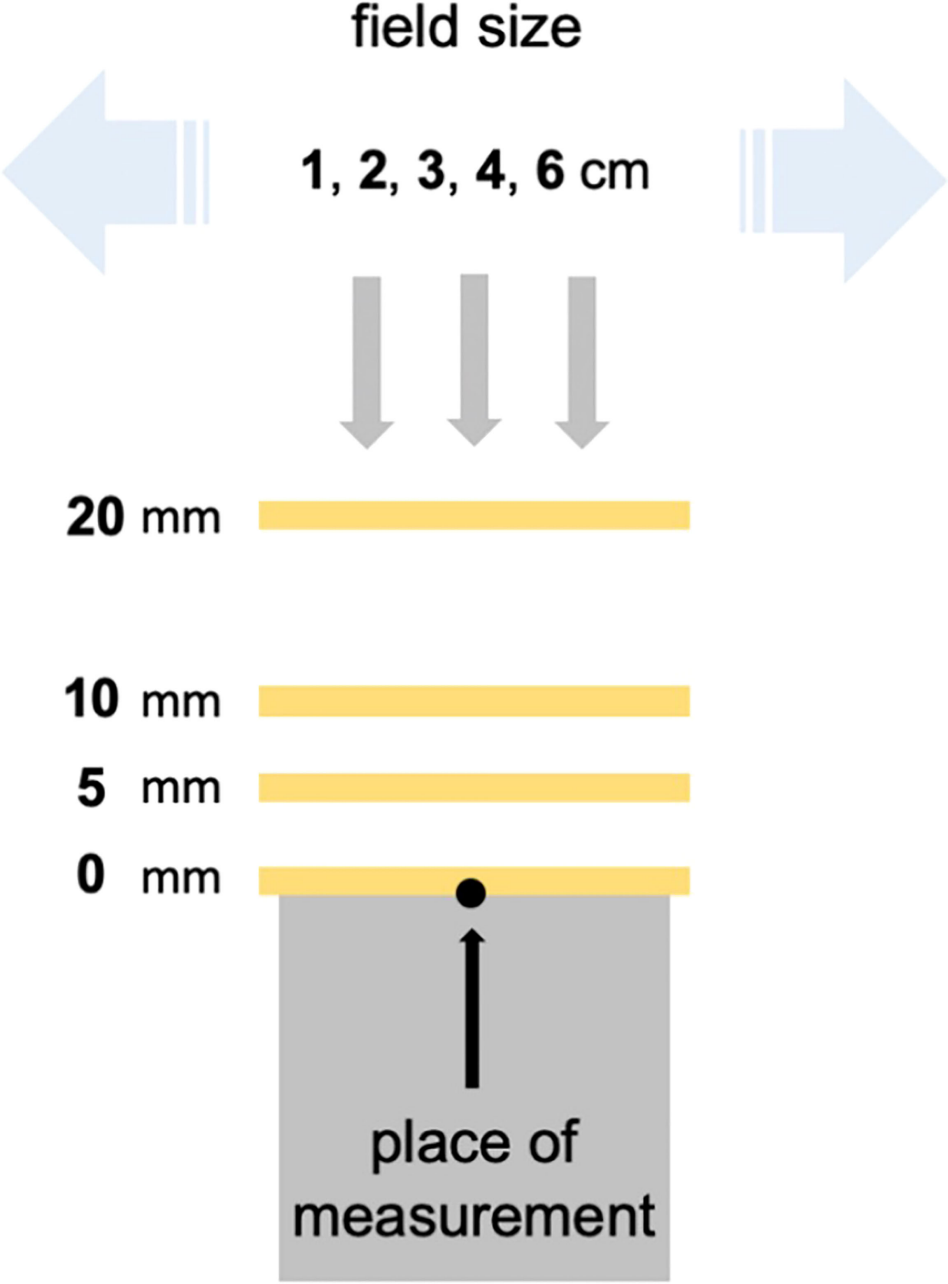
Experimental setup to estimate the influence of distance and field size on dose reduction.

To estimate the influence of surface–bolus distance and field size on the dose reduction, EBT3-film measurements were performed on the surface of a slab phantom. For this purpose, a conventional bolus with a size of 30 × 30 cm^2^ was placed parallel to the slab phantom surface at different heights (0, 5, 10, and 20 mm) and irradiated at different field sizes (1 × 1, 2 × 2, 3 × 3, 4 × 4, and 6 × 6 cm^2^). 3D-printed spacers with very thin support structures of varying heights were used at a distance of 4 cm from the central beam axis for suspension of the boluses. The irradiation was performed in the static field technique with a Siemens linear accelerator (Siemens Primus Mevatron M, 6 MV) with 200 MUs. The experimental setup is shown in **Figure 1**.

### Preparation of the Individual Bolus

First, molds for flabs with edge lengths of 10 × 10 × 0.5 cm were printed, and the dimensions of the casting product were checked for quality assurance.

**Figure 2:**
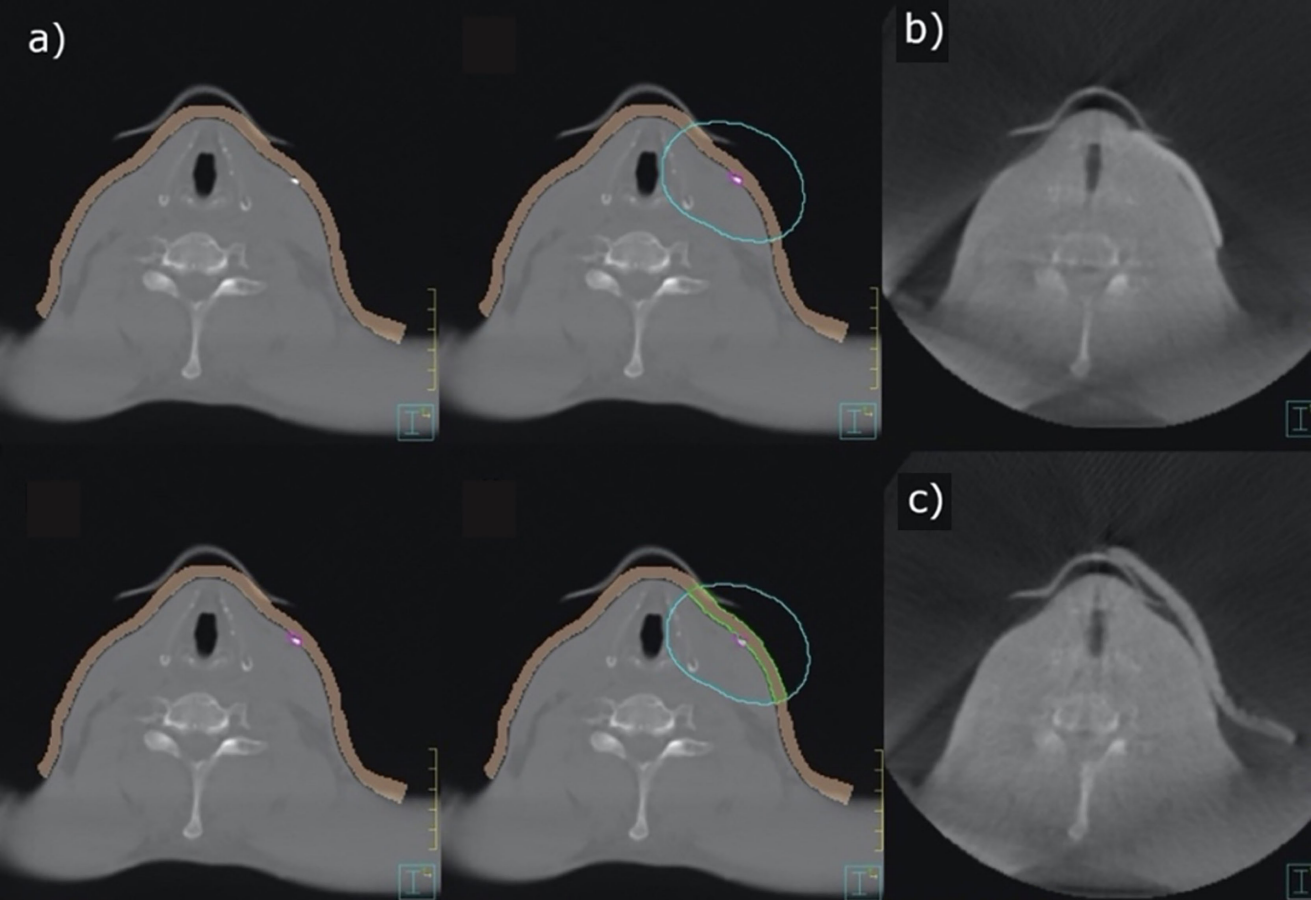
Contouring of an individual bolus **(A)**, 3D-conformal bolus **(B)**, and commercially available bolus **(C)**.

The planning CTs of 25 patients receiving adjuvant radiotherapy of the head and neck were exported to the TPS Pinnacle^®^. An appropriate treatment plan was then determined by the treating radiation oncologist. A virtual 5-mm-thick bolus contour (brown contour, **Figure 2A**) with a virtual density of 1 g cm^−3^ was generated by TPS. The fiducial marking of the target volume (surgical scar) was located and manually drawn along its course for each CT slice (purple contour, **Figure 2A**). The contour indicating the course of the scar was subsequently radially expanded by 2.4 cm to ensure reliable scar coverage (blue contour, **Figure 2A**). With a radius of 2.4 cm around the marker and a CT layer thickness of 3 mm, an even number of layers in the orthogonal direction was obtained. The intersection of the brown and blue volumes was defined as the individual bolus contour (green contour, **Figure 2A**). From this template, a silicone bolus was produced and is shown in **Figure 2B** for comparison. **Figure 2C** shows a commercially available bolus.

**Figure 3:**
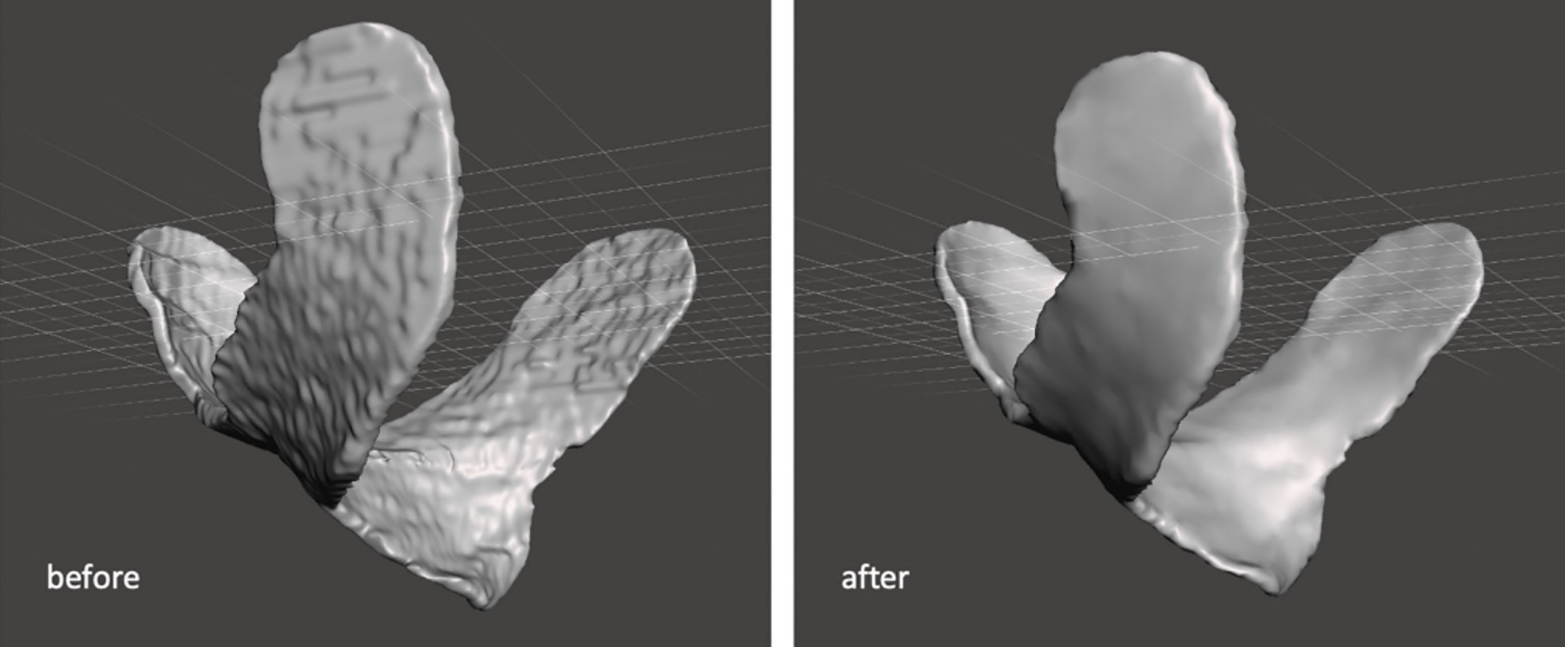
Smoothing the contour of 3D-conformal bolus.

The 3D-conformal bolus contour was exported as a digital imaging and communications in medicine (DICOM) file and converted to standard triangulation language (STL) format using slicing software (3D-Slicer, Version 4.10.2, Slicer Community). This step enabled further processing in 3D printing software (Meshmixer, Version 3.5, Autodesk Inc.). To reduce the time of 3D printing and improve the conformity of the bolus, the surface of the contour was smoothed (**Figure 3**).

The processed and optimized contour was converted into a corresponding casting mold using “Ultimaker Cura” (Version 4.4.1). An automated Cura print profile (**Table 1**) was defined for this application.

**Table 1:** Setting of the automated Cura print profile.

Parameter	Setting
Layer height	0.2 mm
Wall thickness	0.8 mm
Wall line count	2
Top/bottom thickness	0.8 mm
Top/bottom layers	4
Infill density	5%
Infill pattern	Gyroid
Printing temperature	205°C
Build plate temperature	60°C
Enable retraction	Yes
Print speed	70 mm/s
Z hop when retracted	Yes
Enable print cooling	Yes
Fan speed	100%
Build plate adhesion type	Brim
Mold mode	On
Minimal mold width	0.8 mm
Mold roof height	0.8 mm
Mold angle	60°
Supports	Outside only

**Figure 4:**
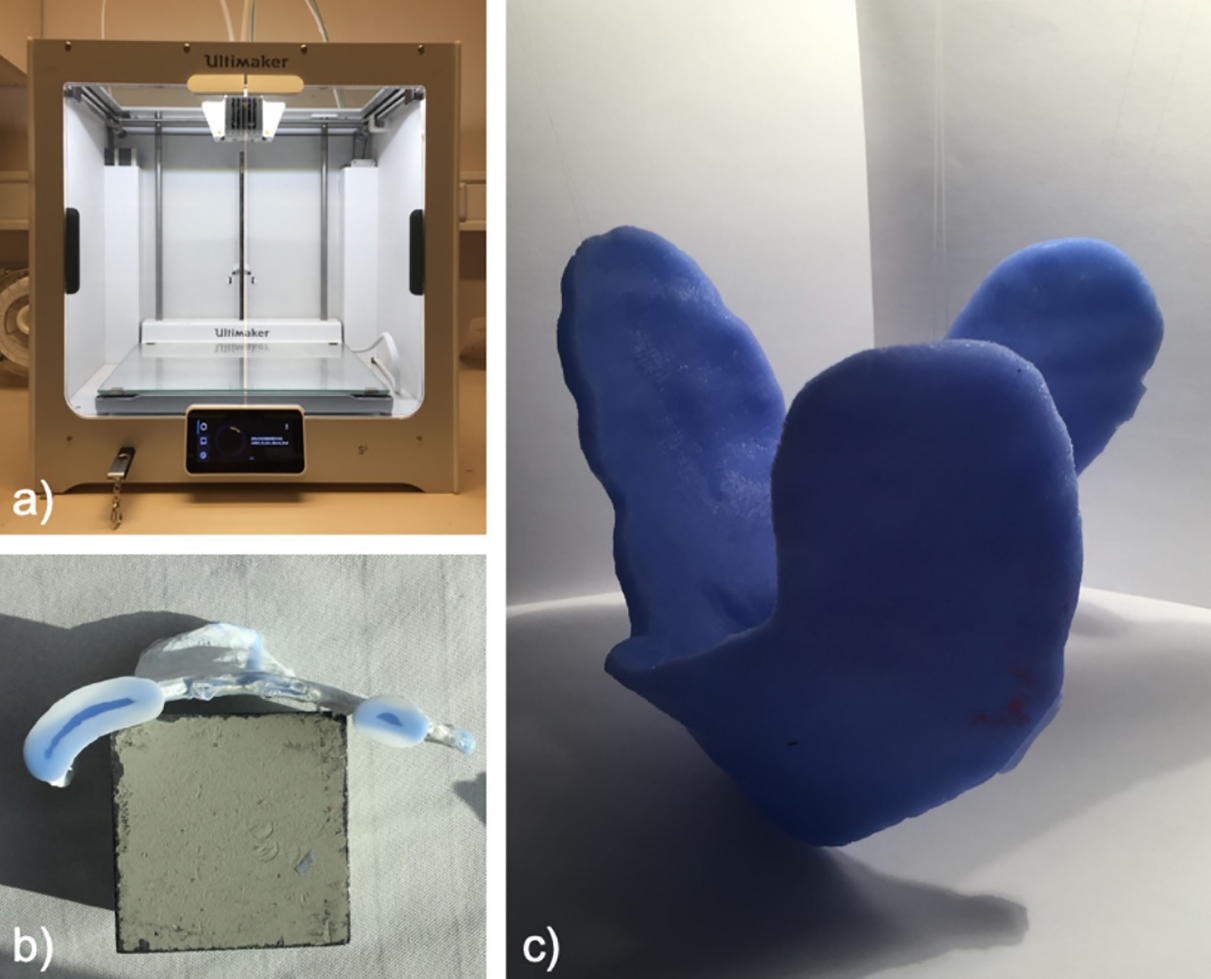
Preparation of the mold with 3D printer **(A)**, mold **(B)**, and individual bolus **(C)**.

Two differently colored crosslinking silicones (Wagnersil 9N, Wagner Dental GmbH & Co. KG) with a density of 1.05 g cm^−3^ and silicone oil (density of 0.97 g cm^−3^) were selected to fill the casting mold. The two silicone components were prepared in a 1:1 ratio. The overall mixing ratio of silicone to silicone oil was 70%:30% v/v due to its ideal softening effect. The mixture was stirred slowly with a glass rod until homogeneous coloration was observed for maximum crosslinking. Afterward, the mixture was filled into the mold, which was printed out of polylactide (PLA NX1, 1.75 mm, white, Extrudr, FD3D GmbH). Air bubbles could be avoided in previous experiments by stirring and filling the silicone slowly. After complete filling of the mold, a minimum time of 30 min was given for the crosslinking reaction before the covering mold was removed (**Figure 4**).

### Dosimetric Investigation of Individual Boluses in Clinical Practice

Three consecutive measurements were made using the commercially available bolus at first and then three more were made subsequently using the 3D-conformal bolus. Both kinds of bolus were placed outside the immobilization mask. Therefore, radiochromic films (8 mm × 10 mm) were placed at three positions located inside the irradiation field. At least one of these was located in the expected high-dose area, directly on the visible surgical scar and preferably in the concave area. Another measurement was taken in the area of healthy skin, which was spared by the individual bolus. The localization of the third position varied depending on the feasibility of these two modalities. These positions were marked with metal pellets; on another day, a cone-beam CT (CBCT) was regularly scheduled. Irradiation of patient plans was performed with an Elekta Versa HD linear accelerator at 6 MV. The single prescribed dose varied from 1.8 to 2.0 to 2.2 Gy (D95) with a simultaneous integrated boost (SIB) according to the risk profile of the corresponding treatment volume.

The films were read out 1 week after the measurement as stated in the manufacturer’s instructions. One CBCT with the commercially available bolus, one CBCT with the 3D-conformal bolus, and the CBCT with the marker were imported into the TPS. The Pinnacle Windowing Protocol “Head” (window, 326; level, 900; L+W, 1226 RAW) was chosen, and the distances between the skin and both boluses were measured at the positions indicated by the markers. All materials and programs are listed in **Table 2**.

**Table 2:** Materials and programs.

Materials and programs	Name of product	Manufacturer
3D printer	Ultimaker S5	Ultimaker B.V., Utrecht, Netherlands
3D printing software	Ultimaker Cura, Version 4.4.1	http://ultimaker.com (Open Source)
Commercially available bolus	Superflab, No. 8117-0.5	Mick Radio-Nuclear Instruments, Inc. An Eckert & Ziegler BEBIG Company, Mount Vernon, USA
Film dosimetry	Gafchromic™ EBT3 Dose range, 0.1 cGy to 10 Gy	Ashland Advanced Materials, Bridgewater, USA
Film dosimetry software	Film QA Pro 2015, Version 5.0.5603.15737	Ashland™ Inc., Covington, USA
Linear accelerator	Siemens Primus Mevatron M 6 MV, Elekta Versa HD™	ELEKTA Instrument AB, Kungstensgatan 18, 113 57 Stockholm, Sweden
Modeling software	Meshmixer, Version 3.5	Autodesk Inc. www.meshmixer.com (Open Source)
MOSFET	MOSFET 20	Thomson & Nielsen Electronics Ltd., Ottawa, Kanada
PLA coil	PLA NX1, 2.85 mm, white	Extrudr, FD3D GmbH, Lauterach, Austria
Slab phantom	RW3 Slab Phantom	PTW-Freiburg, Freiburg im Breisgau, Germany
Therapy planning system	Pinnacle^3®^, Version 16.2	Philips Medical Systems, Hamburg, Germany
Scanner	EPSON^®^ Expression 11000XL	Seiko Epson Corporation, Suwa, Nagano, Japan
Silicone component 1	Wagnersil 9N Premium Dubliersilikon 1:1 Additionsvernetzender RTV-2K Silikonkautschuk	Wagner Dental GmbH & Co. KG, Hückelhoven, Germany
Silicone component 2	Wagnersil 9N Premium Dubliersilikon 1:1 Additionsvernetzender RTV-2K Silikonkautschuk	Wagner Dental GmbH & Co. KG, Hückelhoven, Germany
Slicing software	3D-Slicer, Version 4.10.2	Slicer Community www.slicer.org (Open Source)
Silicone oil	Wagnersil S200 Hochreines, farbloses, geruchloses Silikonöl	Wagner Dental GmbH & Co. KG, Hückelhoven, Germany

The PLA coil in table 1 has a thickness of 2.85 mm.

## Results

### Equivalence of Materials

Doses at the surface of the slab phantom only changed insignificantly under conventional and cast boluses (under conventional bolus, mean = 101.5 cGy, SD = 1.8 cGy; under individual bolus, mean = 103.3 cGy, SD = 1.8 cGy). Both materials could therefore be considered comparable for dose buildup.

### Influence of Field Size and Bolus–Surface Distance on Dose

**Figure 5:**
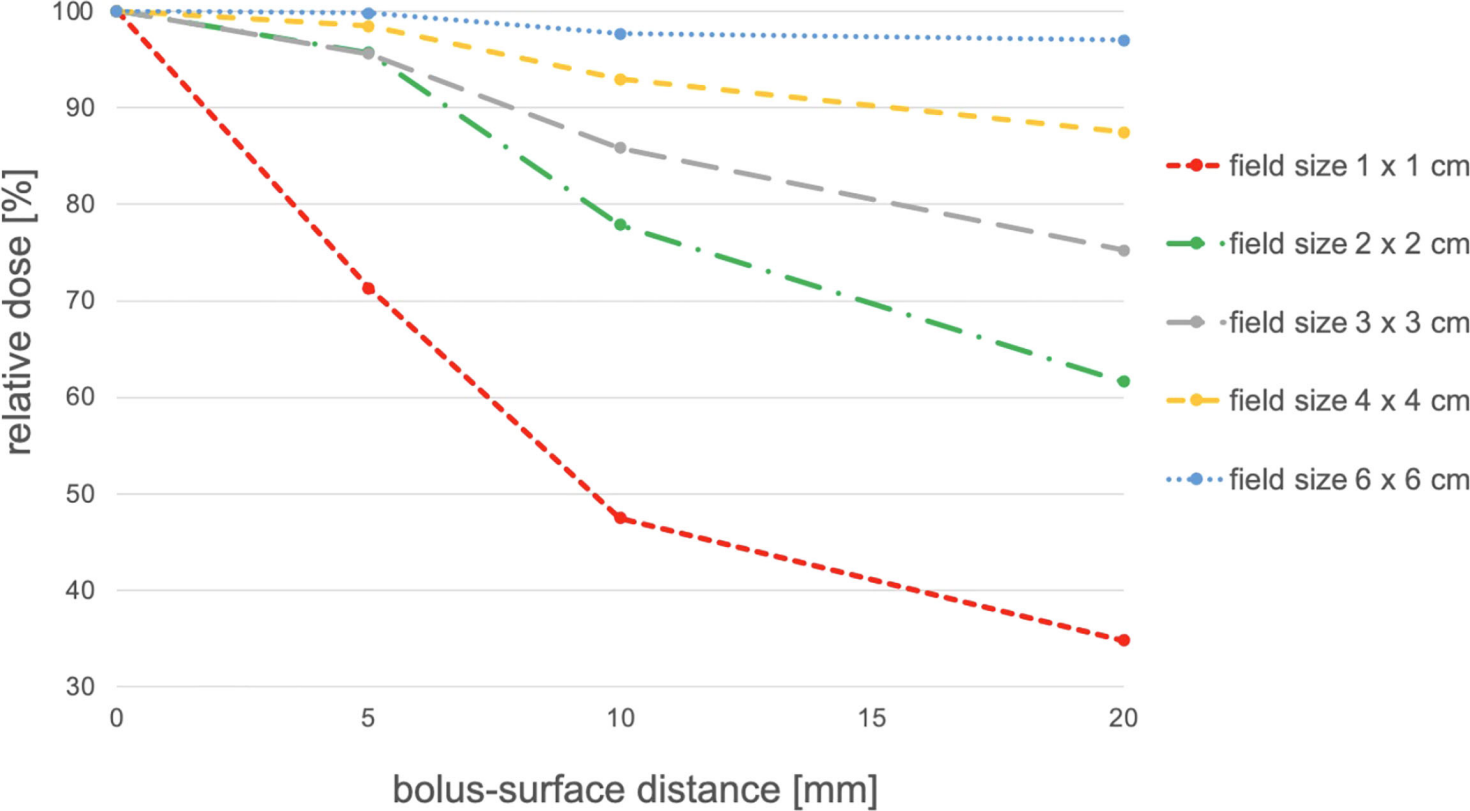
Influence of bolus–surface distance and field size on dose reduction.

The measured values given in **Table 3** as a percentage dose are shown graphically in **Figure 5**. It can be shown that dose reduction due to bolus elevation increases with decreasing field size. While an increase of the surface–bolus distance from 0 to 5 mm still led to a dose reduction of 28.7% for a field size of 1 cm^2^, the dose was reduced by only 4.3% for a field size of 2 cm^2^. Likewise, an increase of the air gap from 0 to 10 mm resulted in a greater relative dose reduction than a further increase from 10 to 20 mm in all cases.

**Table 3:** Relative dose according to field size and surface bolus distance in mm.

Field size [cm × cm]	Absolute and Relative dose at distance0 mm [cGy/%]	Relative dose at distance5 mm [%]	Relative dose at distance10 mm [%]	Relative dose at distance20 mm [%]
**1 × 1**	168.8/100	71.3	47.5	34.9
**2 × 2**	177.2/100	95.7	77.9	61.6
**3 × 3**	194.5/100	95.6	85.8	75.3
**4 × 4**	195.6/100	98.5	93.0	87.5
**6 × 6**	201.3/100	99.8	97.6	97.0

### Dosimetric Evaluation of Individual Boluses

**Figure 6:**
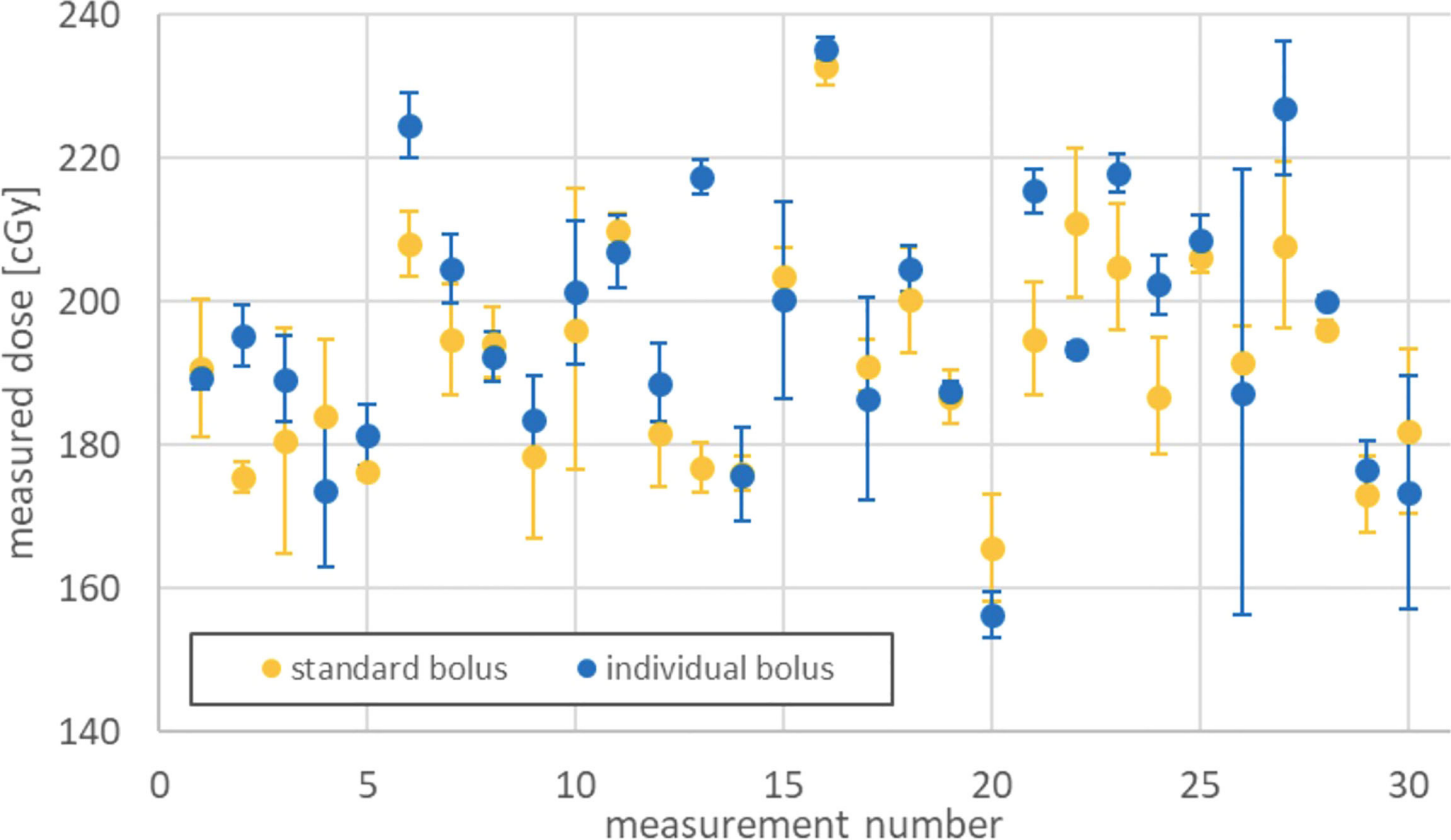
Surface dose under standard and individual boluses.

Across all patients, there were 75 different locations that could be measured. Of these, at a total of 30 different positions, a surface dose could be determined in the scar region under conventional and individual boluses (**Figure 6**). These shown doses are the mean of three single fractions measured by film dosimetry with SDs indicated. The quotient of the surface doses under conventional and individual boluses reveals relative dose increase or decrease. A dependent t-test shows a significant dose difference of +2.45% in the scar region under individual boluses (95%CI 0.0014–0.0477, p < 0.05, N = 30) in comparison to standard ones.

**Figure 7:**
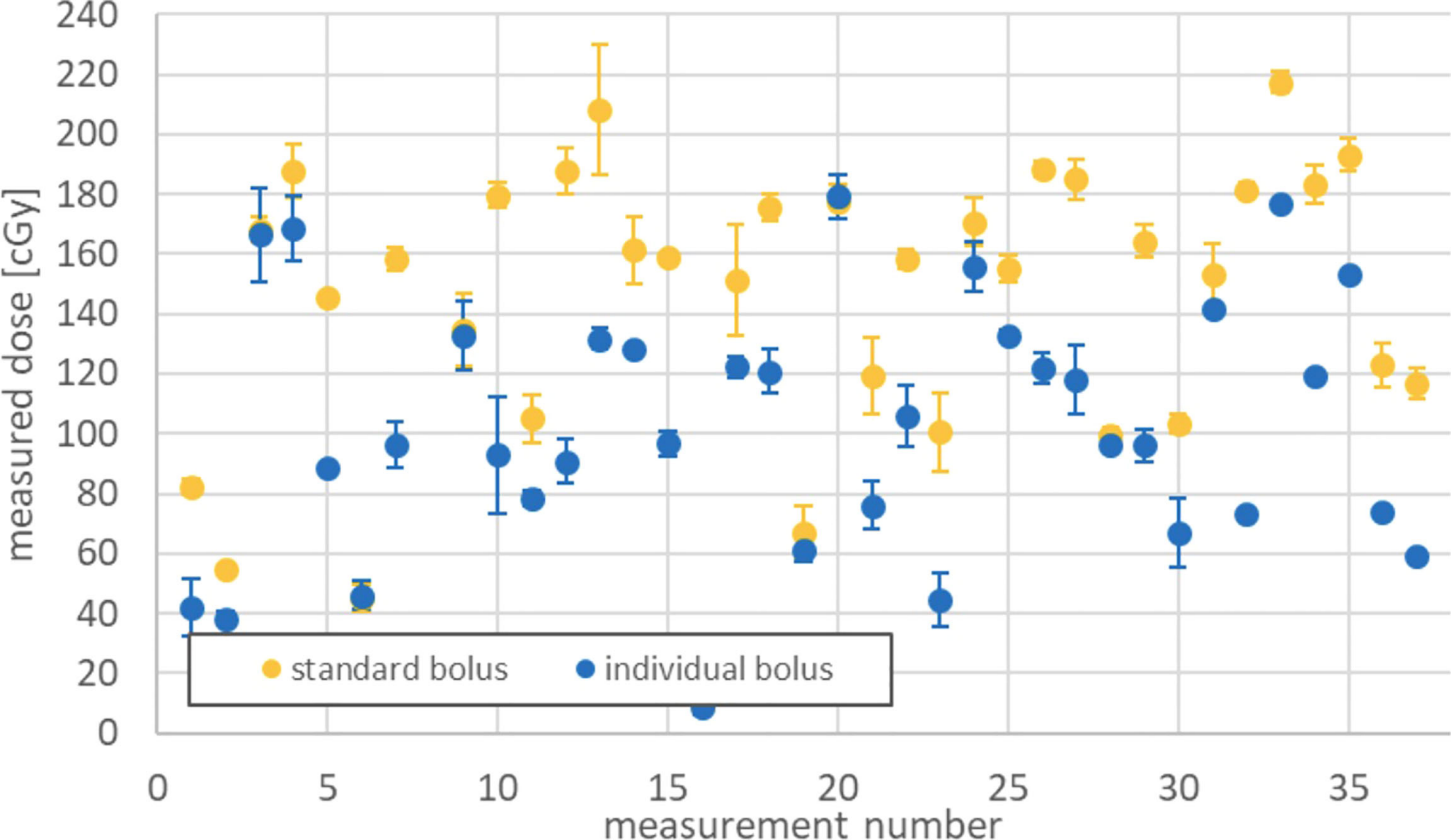
Dose sparing on healthy skin by applying individual boluses.

A total of 37 different positions could be determined at which it was possible to avoid covering healthy skin (sparing) by applying individual boluses (**Figure 7**). The stated doses are the mean value from three single doses as well. A dependent t-test shows a significant dose reduction of 25.9% on skin spared by the omitted bolus coverage (95%CI 19.5–32.3, p < 0.01, N = 37).

At a total of 8 measurement points, neither of the first two criteria was applied, as the measuring point was located under both the individual and conventional boluses but not directly on a scar due to other inadequate attachment options. These points had to be removed from the evaluation.

Surface–bolus distances could be measured at 36 different measurement sites under conventional boluses, while 37 measurement points could be determined under individual boluses.

**Figure 8:**
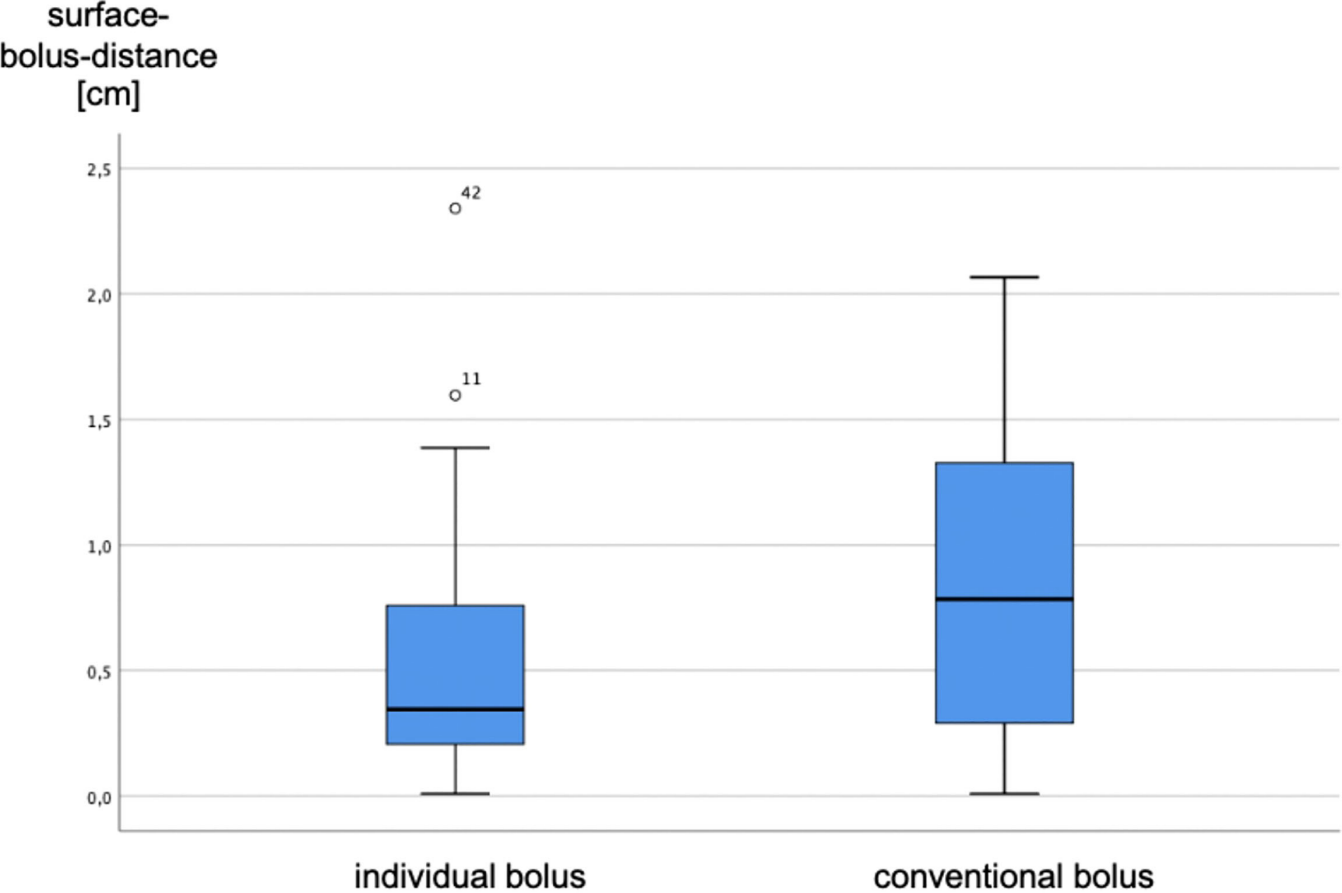
Surface–bolus distances under conventional and individual boluses.

The two populations were compared using a Wilcoxon test on connected samples. The median spacing (50th percentile) under individual and conventional boluses differed significantly (3.5 vs. 7.9 mm, p = 0.001). Thus, individual bolus adjustment resulted in a significant distance reduction (Δ surface–bolus distance). The results are shown in **Figure 8**. Points 11 and 42 represent statistical outliers.

## Discussion

The production of a flexible individual silicone bolus following the suggested procedure was feasible. All boluses endured the mechanical stress during the whole course of the treatment. Measurements on the slab phantom confirmed the dosimetric equivalence of the silicone bolus.

The majority of dose measurements on the scar region are consistent with the expected dose range between the D95 prescription levels of 180 cGy for the lower dose target volume and 220 cGy for the higher dose target volume. Details depend on the location of the individual measurement points in relation to the target volumes and dose distributions. A small but statistically significant increase of the measured dose on the scar region for individual bolus was demonstrated. At the same time, a statistically relevant decrease of the air gap size by 4.4 mm was found. In general, as shown in the slab phantom measurements, the surface dose decreases with increasing gap size. Therefore, the observations seem consistent.

The measured increase in dose buildup may be partly due to the better fit, but irregularities in the bolus thickness may also be a cause. The individual bolus provided approximately 5 ± 0.5 mm of buildup material due to material and thickness accuracy. Therefore, we would expect dose variations of up to 3%, and the measured dose increase of 2.45% is in this range.

In areas of large surface slope gradients (e.g., mandible and neck), the determination of the surface–bolus distance at the point of measurement is challenging. Surface–bolus distances represent an average of measurements from three adjacent CT slices. Further, all stated distance measurements in the transverse plane are performed perpendicular to the patient’s surface. The gap size can differ slightly from the projection of the orthogonal beam direction typically encountered using the VMAT technique. In addition, a field size dependence of the dose reduction at different air gap sizes was observed in the slab phantom.

Omission of unintended skin coverage by bolus resulted in a statistically significant dose reduction of 25.9%. Thus, the resulting dose distributions using individual bolus provided adequate buildup in the scar region and improved sparing of healthy skin.

3D printers are becoming more widespread and affordable for most radiotherapy departments. The cost of such a printer and the personal engagement during the production process of individual bolus must be weighted against the cost of commercial bolus and expected therapy outcome using individual versus commercial bolus. Since unwanted dose discrepancies due to bolus cavities increase with smaller field size, a 3D-conformal adaptation of boluses seemed particularly interesting for VMAT of the head and neck region in which a high number of small field apertures are common.

The use of individual bolus has some limitations in clinical practice. A gapless bolus application is impeded in some areas by the thermoplastic masks used for immobilization, which causes some cavities themselves. Additionally, even for an initially perfectly fitting bolus, further gaps may arise during the course of the treatment by treatment-induced changes in the irradiated target volume, such as a decrease in swelling in the head and neck region or changes in position during mask fitting. Due to the first problem, we plan to use a 3D surface scanner to manufacture an individual bolus to be fitted under the thermoplastic mask. This allows full integration of individual bolus into the immobilization mask. The dose buildup effect caused by the masks, which was absent at measurement points outside the mask fixation, may bias the conclusion of a purely bolus-related dose buildup.

## Conclusion

Individual boluses *via* FDM in conjunction with silicone casting are possible and practicable. The individual steps required were optimized with regard to the virtual generation of the bolus contour from the TPS, the production of a mold using 3D modeling software, and the mixing ratios of the silicone casting components. The boluses did tolerate the mechanical stresses over the entire treatment period.

Measurements comparing individual and conventional bolus variants showed a slight but significant increase in surface dose in the critical scar region in favor of individual ones. The custom manufacturing process significantly reduced unwanted dose exposure to healthy skin with a dose decrease of 25.9%. Likewise, a better fitting of the customized bolus was evident with a significant reduction in the surface–bolus distance (3.5 vs. 7.9 mm).

## Data Availability Statement

The original contributions presented in the study are included in the article/**Supplementary Material**. Further inquiries can be directed to the corresponding author.

## Ethics Statement

The studies involving human participants were reviewed and approved by the Ethics Committee of the University Hospital Würzburg (Director: Prof. Dr. Roland Jahns). The patients/participants provided their written informed consent toparticipate in this study.

## Author Contributions

SP, VL, MF, AT, and SW designed the study. SP, SW, and VL were involved in the data collection and analysis. All authors critically reviewed and gave final approval of the manuscript.

## Funding

This publication was supported by the Open Access Publication Fund of the University of Würzburg.

## Conflict of Interest

The authors declare that the research was conducted in the absence of any commercial or financial relationships that could be construed as a potential conflict of interest.

## Publisher’s Note

All claims expressed in this article are solely those of the authors and do not necessarily represent those of their affiliated organizations, or those of the publisher, the editors and the reviewers. Any product that may be evaluated in this article, or claim that may be made by its manufacturer, is not guaranteed or endorsed by the publisher.

## Abbreviations

CBCT, cone-beam CT; DICOM, digital imaging and communications in medicine; FDM, fused deposition modeling; HUs, Hounsfield units; IMRT, intensity-modulated radiotherapy; MU, monitor units; STL, standard triangulation language; TPS, therapy planning system; VMAT, volumetric modulated arc therapy.
